# Tribological Behavior of Aluminum Hybrid Nanocomposites Reinforced with Alumina and Graphene Oxide

**DOI:** 10.3390/ma15030865

**Published:** 2022-01-23

**Authors:** Abdul Samad Mohammed, Omar Saad Aljebreen, Abbas Saeed Hakeem, Tahar Laoui, Faheemuddin Patel, Mirza Murtuza Ali Baig

**Affiliations:** 1 Department of Mechanical Engineering, King Fahd University of Petroleum and Minerals, Dhahran 31261, Saudi Arabia; g201519830@kfupm.edu.sa (O.S.A.); faheemmp@kfupm.edu.sa (F.P.); mmurtuza@kfupm.edu.sa (M.M.A.B.); 2 Interdisciplinary Research Center for Advanced Materials, King Fahd University of Petroleum and Minerals, Dhahran 31261, Saudi Arabia; 3 Interdisciplinary Research Center for Hydrogen & Energy Storage (IRC-HES), King Fahd University of Petroleum & Minerals, Dhahran 31261, Saudi Arabia; ashakeem@kfupm.edu.sa; 4 Department of Mechanical and Nuclear Engineering, University of Sharjah, Sharjah P.O. Box 27272, United Arab Emirates; tlaoui@sharjah.ac.ae

**Keywords:** aluminum hybrid composites, alumina, graphene oxide, tribology

## Abstract

Due to rapid technological advancements, the demand for lightweight materials with improved tribo-mechanical properties is continuously growing. The development of composite materials is one of the routes taken by researchers to meet these target properties. Aluminum (Al) is one of the most suitable materials used for developing composites for a wide range of applications because of its light weight, high conductivity, and high specific strength. In this study, aluminum hybrid nanocomposites with alumina (10 Vol% Al_2_O_3_) and varying loadings of graphene oxide (0.25, 0.5 and 1 wt% GO) were fabricated using the spark plasma sintering technique. The tribological properties of the developed hybrid composites were evaluated by conducting ball-on-disk wear tests at a normal load of 3N, with a sliding speed of 0.1 m/s, and for a sliding distance of 100 m. A 440C hardened stainless steel ball with a diameter of 6.3 mm and a hardness of 62 RC was used as a counterface. Scanning electron microscopy (SEM), elemental X-ray dispersive analysis (EDS), and optical profilometry were used to ascertain the involved wear mechanisms. The results revealed that Al-10 Vol%Vol% Al_2_O_3_-0.25 wt% GO hybrid nanocomposite showed an increase of 48% in the hardness, a reduction of 55% in the specific wear rate, and a reduction of 5% in COF compared with pure aluminum.

## 1. Introduction

The demand for lightweight and high strength materials has been on the rise, primarily due to an expansion of the aerospace and automobile industries [1,2,3,4,5]. Metallic materials are used extensively in various engineering applications for their excellent mechanical and thermal properties. However, they suffer from two major drawbacks, such as high wear and low corrosion resistance. Hence, as the world is moving towards more industrialization, an emphasis on energy conservation has led to many researchers looking for avenues to further improve the properties of metals with a special focus on improving the tribological and anti-corrosive properties of metals. However, the inadequacy of metals and alloys in providing both high strength and stiffness required for demanding engineering applications, coupled with two major drawbacks such as high wear and low corrosion resistance, has led to the development of metal matrix composites (MMCs). Metal matrix composites can be designed to possess qualities such as a low coefficient of friction, low wear, and good anti-corrosive properties, making them suitable for use in demanding engineering applications [6].

Aluminum is one of the lightweight materials extensively studied over the years because of its excellent thermal conductivity and high strength-to-weight ratio [7]. However, the use of pure Al is generally limited to simple engineering applications, which necessitated the development of aluminum-based alloys with enhanced mechanical, corrosion, and tribological properties for a wider range of applications [8,9,10,11]. Further improvements to the properties of aluminum-based materials were achieved through the development of aluminum metal-based matrix composites (Al-MMCs) by adding different reinforcements to achieve tailored property combinations [12,13]. Ceramic reinforcements such as silicon carbide [14,15,16], titania [17], alumina [18,19,20,21,22], a combination of different ceramics [23], and carbon-based materials such as carbon fibers [24,25], graphite [26,27], carbon nanotubes (CNTs) [28,29], graphene [30,31], and graphene oxide (GO) [32,33] were used as reinforcements to fabricate Al-MMCs with improved properties.

Aluminum possesses high corrosion resistance under the majority of service conditions but has inferior resistance to wear. This has prompted researchers to develop Al-MMCs with improved tribological properties by incorporating different ceramic and carbon-based reinforcements by employing various fabrication methods. Irrespective of the fabrication process, tribological properties of Al-MMCs with single reinforcement showed improvement over pure aluminum and its alloys, and hybrid reinforcement demonstrated better tribological properties over single reinforcement [34]. Al-MMCs prepared by incorporating 5 wt% of B_4_C particles as reinforcement using the stir casting method showed improved tribological properties. Sliding wear rate and coefficient of friction were found to increase with the load but decrease with velocity and sliding distance [35]. Stir cast Al-MMCs prepared with 0.5, 1, 1.5, 2 and 2.5 wt.% nano silicon carbide powder as reinforcement showed a reduction in wear rate and friction coefficient with increasing SiC content. This was attributed to the increase in hardness and the lubricating nature of the SiC particles that became detached and polished the worn-out surface of the samples [36]. Wear behavior of Al_2_O_3_ nanoparticles reinforced Al-MMCs prepared using the ultrasonic-assisted semisolid stirring (UASS) method showed a progressive decrease in wear rate with an increase in reinforced Al_2_O_3_ content from 1% to 7%; however, wear rate increased with 10% Al_2_O_3_ content, which was attributed to an increase in agglomeration of Al_2_O_3_ nanoparticles [37]. A similar improvement in the wear resistance was observed in studies with Al_2_O_3_ and ZrO_2_ nanoparticles as reinforcements to Al-MMCs prepared using uniaxial compaction followed by sintering. The improvement in wear resistance correlated with the increase in hardness, owing to the hardness of the ceramic particle reinforcements [38,39]. The effect of hybrid ceramic reinforcements on the tribological behavior of Al-MMCs was also investigated. Hybrid SiC-TiC-Al-MMCs prepared using liquid stir casting process showed an improvement in wear resistance with an increase in the TiC reinforcement content from 1 to 2.5 wt%, whereas SiC content was fixed at 1 wt% [40]. Al-MMCs prepared using carbon-based materials as reinforcements were shown to affect tribological behavior. Al-MMCs prepared using the spark plasma sintering (SPS) process with 2 Vol%Vol% graphene nanoplatelets (GNPs) showed a lower coefficient of friction but increased wear rate under room temperature and at 200 °C wear testing. The decrease in the coefficient of friction was attributed to the slippage of interlayers of GNP. In contrast, the poor wear resistance was attributed to the agglomeration of GNPs, which resulted in poor densification and insufficient sintering [41]. However, it was found that the addition of graphite particles as a reinforcement to SiC-graphite-reinforced hybrid Al-MMCs led to the reduction in friction coefficient and frictional heat produced during sliding wear due to the formation of solid lubrication film [42]. The addition of ceramic reinforcements to Al-MMCs was shown to affect the corrosion behavior as well. Al-MMCs prepared with SiC reinforcements produced by uniaxial compaction and hot extrusion showed improved corrosion resistance upon increasing SiC content and was attributed to the chemical inertness of SiC particles to a corrosive solution. Moreover, SiC-Al-MMCs exhibited higher wear resistance and lower friction coefficients when wear-tested under corrosive media as compared with pure aluminum [43].

From the above literature, it is clear that the addition of ceramic reinforcement, as well as the addition of carbon reinforcement, tends to affect the tribological behavior of the developed Al-MMCs. Moreover, the type of processing method affects the developed properties. Few studies were reported on aluminum composites reinforced with either Al_2_O_3_ or graphene nanoplatelets individually. The present work reports on the synthesis, mechanical properties, and tribological behavior of aluminum hybrid nanocomposites reinforced with Al_2_O_3_ and GO, prepared using the spark plasma sintering process.

## 2. Materials and Methods

Aluminum (Al) powder from the Alpha chemical company, Dartmouth, Canada, with a purity of 99.5% and an average particle size of 30 µm was used as the matrix material. Aluminum oxide (Al_2_O_3_: Purity of 99.88%, particle size of 300 nm, approximate surface area of 85–115 m^2^/g) was procured from Union Carbide Corporation for BUEHLER Ltd. Lake Bluff, IL, USA and used as the first reinforcement. Graphene oxide (GO) from the AD Nano Company, Shimoga, India, with 99% purity and surface area of 250 m^2^/g was used as the second reinforcement.

### 2.1. Fabrication of the Hybrid Nanocomposites

In a previous study [44], the Al_2_O_3_ content in the Al matrix in terms of density and hardness and GO content in terms of mechanical and thermal properties was comprehensively optimized. It was observed that Al-10 Vol%Vol% Al_2_O_3_ resulted in the highest hardness of 55.8 HV compared with the other tested loadings of 20 and 30 Vol%Vol%, respectively. The improvement in the properties of Al-10 Vol%Vol% Al_2_O_3_ was attributed to the uniform dispersion of Al_2_O_3_ within the Al matrix, as observed from the scanning electron microscopy images. Moreover, among the hybrid nanocomposites, Al-10 Vol% Al_2_O_3_-0.25 wt% GO exhibited the highest hardness of 63 HV, the highest compressive strength of 180 MPa, and the lowest thermal expansion of 14.82 ppm °C^−1^ compared with the pristine and Al-10 Vol% Al_2_O_3_ samples. Since the mechanical and thermal properties of the Al-10 Vol% Al_2_O_3_-X wt% GO hybrid nanocomposites were comprehensively evaluated in [44], the aim of the present study was defined as the evaluation of tribological properties of Al-10 Vol% Al_2_O_3_-X wt% GO, wherein X represents the different GO loadings (0.25, 0.5 and 1 wt%) in the hybrid nanocomposite. The detailed procedure of preparing the hybrid nanocomposites, including the ball milling parameters and the spark plasma sintering parameters, can be found in the earlier research [44] and is also described below briefly.

#### 2.1.1. Sonication and Ball Milling to Disperse and Mix the Powders

To obtain a uniform dispersion, 10 Vol% Al_2_O_3_ nanoparticles were sonicated in ethanol for 1 h using a probe sonicator (Sonics VCX 750, Newtown, CT, USA) with a cycle amplitude of 45% (ON: 20 s/Off 5 s) at room temperature. The same procedure was repeated for breaking the agglomeration in GO. The dispersed Al_2_O_3_ was then added to the weighed quantity of Al powder for ball milling (HD/HDDM/01, Union process, Inc., Akron, OH, USA) in zirconia vials. Ball milling was carried out for 24 h at a rotational speed of 200 rpm, using zirconia balls (diameter = 5 mm) with a ball to powder ratio of 10:1 for homogeneous mixing. The milling process was carried out in an argon atmosphere to avoid oxidation of the powders and in the presence of 50 mL of ethanol to avoid any cold welding during the process. The mixed powders were then dried in an oven at a temperature of 80 °C for 12 h to evaporate ethanol completely. To prepare the hybrid powders, GO was added in different percentages of 0.25, 0.5, and 1 wt%, respectively, to the mixture of Al-10 Vol% Al_2_O_3_ and mixed for 24 h with the same milling parameters.

#### 2.1.2. Spark Plasma Sintering Process

Circular samples of 20 mm diameter and a thickness of 6 mm for each formulation were produced using spark plasma sintering (SPS) equipment (SPS FCTsystem GmBH, Frankenblick, Germany) under a high vacuum. Table 1 shows the SPS parameters used for fabricating the samples.

### 2.2. Densification and Hardness Measurements

The density of the fabricated samples was measured by immersing the sample in deionized water according to Archimedes principles and rule of mixtures by using (Kern ABT weighing scale, 320 g capacity, Nordrhein-Westfalen, Germany). The hardness was measured using a Zwick hardness tester by using an applied load of 500 gf. An average of 10 readings is reported for each sample.

### 2.3. Friction and Wear Tests

Ball on disk wear experiments were conducted using a tribometer (UMT-3, Bellerica, MA, USA) to determine the friction and wear characteristics of the composites. The counterface used was a 440C stainless steel ball with a diameter of 6.3 mm and a hardness of 62 RC. Wear tests were conducted at a normal load of 3 N, with a sliding peed of 0.1 m/s for 5000 cycles corresponding to a sliding distance of 100 m. The wear volume loss was estimated using a 3D optical profilometer (GTK-A, Bruker, Bellerica, MA, USA). A scanning electron microscope (SEM: Tescan VEGA^3^, Brno, Czech Republic) with an electron X-ray dispersive (EDS) attachment was used to ascertain the involved wear mechanisms and the role of the wear debris particles. An optical microscope was used to record the counterface ball images to evaluate its wear.

## 3. Results

### 3.1. Density and Hardness of the Nanocomposites

Table 2 shows the densification of all the formulations, measured based on the Archimedes method. It was observed that the density of the samples reduced marginally with the addition of the reinforcements, with pure aluminum showing the highest density of 99.8% compared with the other compositions.

**Figure 1:**
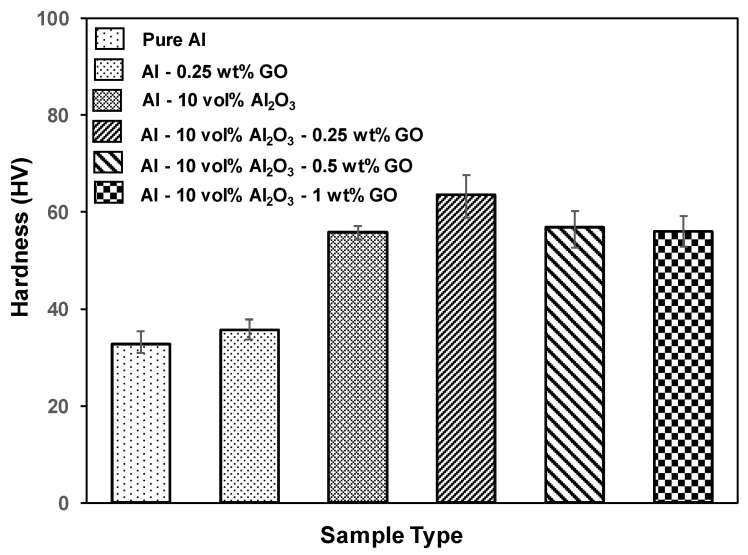
Variation of the hardness of Al nanocomposite and hybrid nanocomposites.

Figure 1 shows the hardness variation with the addition of Al_2_O_3_ and GO to the Al matrix. It can be observed from Figure 1 that the hardness increased significantly with the addition of Al_2_O_3_ from 32.80 HV to 55.83 HV. This is attributed to the inherent hardness of the Al_2_O_3_ particles and their uniform dispersion within the Al matrix, as reported in the earlier study [44]. Moreover, with the addition of 0.25 wt% of GO to the Al-10 Vol% Al_2_O_3_ composite, the hardness increased to 63.57 HV, after which the hardness decreased with increasing GO content. The increase in hardness with the addition of 0.25 wt% GO was attributed to the uniform dispersion of both fillers within the aluminum matrix without the formation of agglomerates. Moreover, evidence from the SEM images of the microstructure analysis of Al-10 Vol% Al_2_O_3_-0.25 wt% GO showed a very fine grain size which contributed to the increase in the hardness. However, the reduction in hardness of the hybrid composites with an increase in the content of GO was attributed to the formation of agglomerates and lower densification of the samples (Table 2) [44]. The variation of hardness with the addition of only GO and Al_2_O_3_ can also be seen from Figure 1. Clearly, the addition of 0.25 wt% GO resulted in a marginal increase in hardness compared with the addition of Al_2_O_3_ nanoparticles. This indicates that Al_2_O_3_ reinforcement is the main contributor for the improvement in the hardness of the hybrid nanocomposite.

### 3.2. Tribological Characterization of the Nanocomposite/Hybrid Samples

#### 3.2.1. Specific Wear Rate of the Developed Nanocomposite/Hybrid Samples

**Figure 2:**
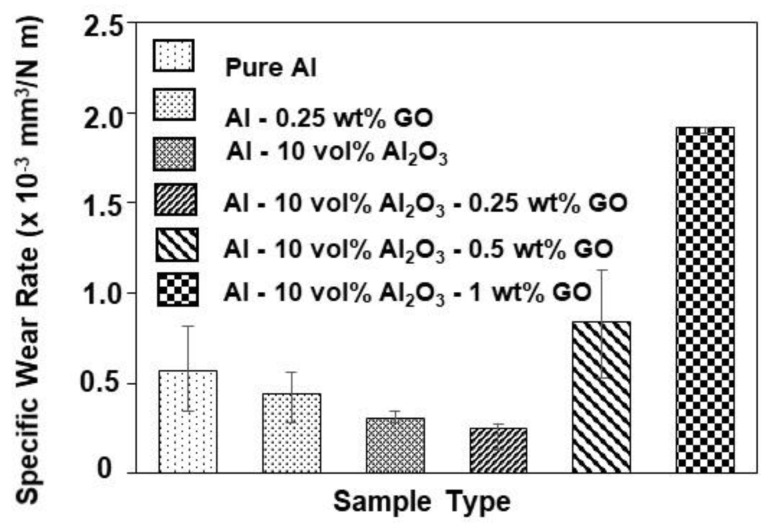
Variation of specific wear rate of different samples at a load of 3 N, sliding speed of 0.1 m/s, and a sliding distance of 100 m.

**Figure 3:**
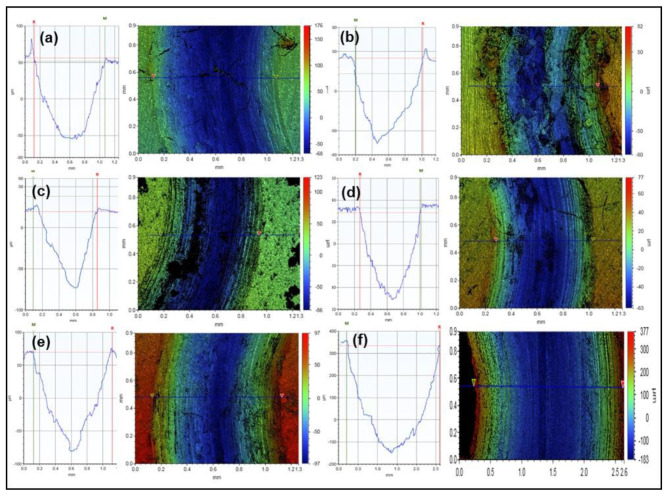
Typical 2D profiles for wear tracks after the wear test at a load of 3 N, sliding speed of 0.1 m/s and a sliding distance of 100 m: (**a**) Pure Al; (**b**) Al-0.25 wt% GO; (**c**) Al-10 Vol% Al_2_O_3_; (**d**) Al-10 Vol% Al_2_O_3_-0.25 wt% GO; (**e**) Al-10 Vol% Al_2_O_3_-0.5 wt% GO; (**f**) Al-10 Vol% Al_2_O_3_-1 wt% GO.

Figure 2 shows the variation in the specific wear rate of the various samples tested under an applied load of 3 N and a sliding speed of 0.1 m/s for a duration of 5000 cycles corresponding to a sliding distance of 100 m. The specific wear rate was calculated by the ratio of the total wear volume loss to the product of sliding distance and the applied load. The wear volume loss was estimated by the product of the wear area, measured by analyzing the 2D profiles of the wear tracks recorded by using a 3D optical profilometer and the circumference of the wear track. Figure 3 shows a few typical 2D profiles as measured by the 3D optical profilometer for each tested sample. According to Figure 2, the specific wear rate of the aluminum composites (Al-0.25 wt% GO and Al-10 Vol% Al_2_O_3_) decreased as compared to pure aluminum, noting that the reduction in the specific wear rate for Al-10 Vol% Al_2_O_3_ was greater than that of Al-0.25 wt% GO. This could be attributed to the higher hardness of the Al-10 Vol% Al_2_O_3_ composite compared with that of the Al-0.25 wt% GO composite, as reported in Figure 1.

For the hybrid nanocomposites, the specific wear rate decreased slightly further upon adding 0.25 wt% GO to Al-10 Vol% Al_2_O_3_, owing to the improved hardness of the hybrid nanocomposite (Figure 1), mainly due to the hard alumina nanoparticles, and to the lubricious nature of GO. Moreover, as reported earlier [44], for the hybrid nanocomposite Al-10 Vol% Al_2_O_3_-0.25 wt% GO, uniform dispersion of both reinforcements within the aluminum matrix was observed, leading to an effective load transfer, which reults in an improvement in the wear resistance. However, as the content of GO increased (0.5 and 1 wt%), the specific wear rate of the hybrid nanocomposites increased significantly. This could be attributed to the reduction in hardness of the samples (Figure 1), lower densification (Table 1), and as reported earlier [44], the formation of agglomerates of the reinforcements within the aluminum matrix.

#### 3.2.2. Friction Coefficient of the Developed Nanocomposites/Hybrid Samples

**Figure 4:**
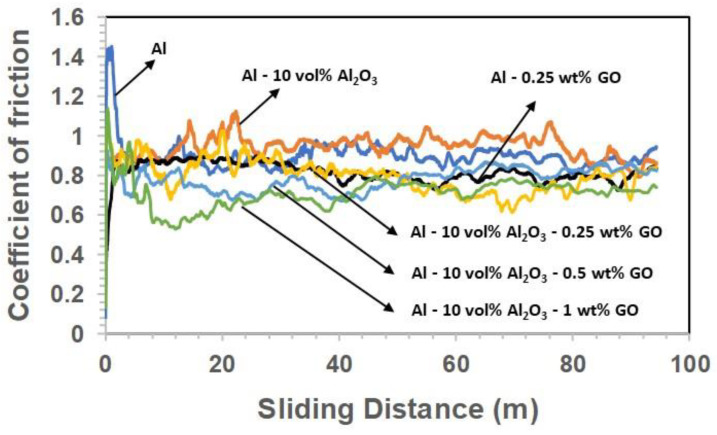
Typical frictional graphs for all the tested samples at a load of 3 N, sliding speed of 0.1 m/s, and a sliding distance of 100 m.

**Figure 5:**
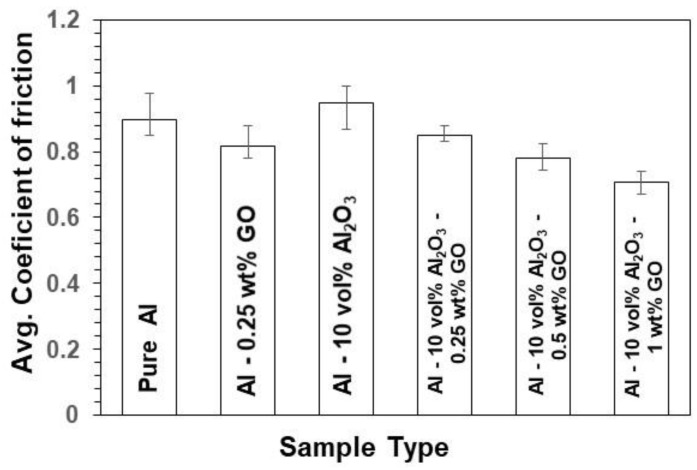
Variation of average coefficient of friction for all the tested samples at a load of 3 N, sliding speed of 0.1 m/s, and a sliding distance of 100 m.

Figure 4 and Figure 5 show typical frictional graphs for the wear tests and the average coefficient of friction (COF) for all tested samples under a normal load of 3 N, a sliding speed of 0.1 m/s, and a sliding distance of 100 m. According to Figure 5, the average COF increased slightly for the Al-10 Vol% Al_2_O_3_ nanocomposites compared with pure Al, mainly due to the presence of the hard alumina particles. Moreover, the COF of Al-0.25 wt% GO was less than that of pure Al and the Al-10 Vol% Al_2_O_3_ nanocomposite, due to the lubricious nature of GO. However, for the hybrid nanocomposite, Al-10 Vol% Al_2_O_3_-0.25 wt% GO, the COF increased slightly as compared with the Al-0.25 wt% GO nanocomposites, due to the addition of hard alumina particles. The COF for the Al-10 Vol% Al_2_O_3_-0.25 wt% GO hybrid nanocomposite was lower than pure Al and Al-10 Vol% Al_2_O_3_ nanocomposites, due to the presence of lubricious GO. In fact, a further increase in the GO content led to a further decrease in COF. The main reason for adding GO to the hybrid nanocomposite was to reduce the COF, as confirmed by these results.

#### 3.2.3. SEM/EDS Analysis

**Figure 6:**
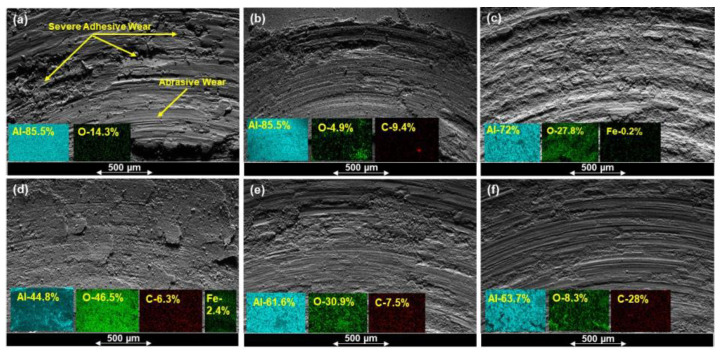
Typical SEM images and EDS mapping for the wear tracks of (**a**) Pure Al (**b**) Al-0.25% wt% GO (**c**) Al-10 Vol% Al_2_O_3_ (**d**) Al-10 Vol% Al_2_O_3_-0.25 wt% GO (**e**) Al-10 Vol% Al_2_O_3_-0.5 wt% GO (**f**) Al-10 Vol% Al_2_O_3_-1 wt% GO, after the wear test at a load of 3 N, sliding speed of 0.1 m/s, and a sliding distance of 100 m.

**Figure 7:**
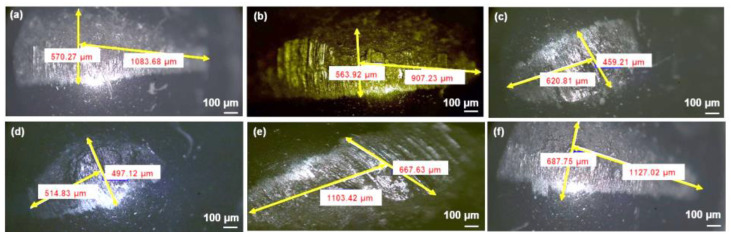
Typical optical counterface ball images sliding against: (**a**) Pure Al (**b**) Al-0.25 wt% GO (**c**) Al-10 Vol% Al_2_O_3_ (**d**) Al-10 Vol% Al_2_O_3_-0.25 wt% GO (**e**) Al-10 Vol% Al_2_O_3_-0.5 wt% GO (**f**) Al-10 Vol% Al_2_O_3_-1 wt% GO, after the wear at a load of 3 N, sliding speed of 0.1 m/s, and a sliding distance of 100 m.

Figure 6 shows the SEM/EDS images of the wear tracks, and Figure 7 shows the optical images of the counterface ball after sliding against the tested samples at a load of 3 N, sliding speed of 0.1 m/s, and for a sliding distance of 100 m.

Pure Al displayed a high wear rate, as shown in Figure 2, and was confirmed by SEM image of the wear track shown in Figure 6a. It shows a very rough surface characterized by deep grooves and debris particles resulting from the ploughing of the surface during the wear test involving a two-body and a three-body abrasive wear mechanism coupled with severe adhesive wear (gouging) mechanism (Figure 6a). The high COF of 0.9 shown by Al (Figure 5) was attributed to the high adhesive and the deformation components of the friction coefficient. The counterface ball sliding against the samples also showed a large scar mark, as shown in Figure 7a.

However, on the addition of 0.25 wt% GO alone to the Al matrix, a reduction of 22% and 9% in the specific wear rate and the COF was observed, respectively. Figure 6b shows a smoother wear track as compared with the pure Al, with a few instances of adhesive wear along the edges. An increase in hardness was observed upon adding 0.25 wt% GO to Al matrix (Figure 1), which could be the reason for its better tribological performance in terms of increased wear resistance. The inherent lubricating properties of GO must have contributed to the reduction in the COF as compared with Al. Moreover, the counterface ball sliding against the Al-0.25 wt% GO also showed a scar mark of similar size, as shown by the ball sliding against the Al sample.

The addition of 10 Vol% Al_2_O_3_ alone to Al matrix resulted in a significant increase in the hardness, as shown in Figure 1. The Al-10 Vol% Al_2_O_3_ composite showed a significant decrease of 46% in the specific wear rate, accompanied by a 6% increase in the COF as compared with Al (Figure 2 and Figure 5). A close examination of the wear track showed the wear mechanism to be mostly abrasive in nature, with ploughing marks visible in the direction of sliding. Small traces of iron were detected on the wear track, which could have been transferred from the counterface ball during the test (Figure 6c). The scar mark on the counterface ball was slightly smaller (Figure 7c) compared with the one observed in the earlier cases. Thus, it can be inferred that the addition of Al_2_O_3_ to Al matrix contributed mainly to the improvement of the hardness of Al, leading to a significant decrease in the specific wear rate.

Upon adding both reinforcements to Al matrix to form a hybrid nanocomposite, an interesting trend was observed. A simultaneous addition of 10 Vol% Al_2_O_3_ and 0.25 wt% GO to Al resulted in an increase of 48% in the hardness, reduction of 55% in the specific wear rate and a reduction of 5% in COF compared with Al. Moreover, this hybrid nanocomposite exhibited a better wear resistance than Al-0.25 wt% GO and Al-10 Vol% Al_2_O_3_ composites (Figure 2). The SEM image analysis of the wear track revealed a smooth surface with traces of wear predominantly by plastic deformation. Moreover, the scar mark on the counterface ball was also relatively small compared with the other samples (Figure 7d). This excellent performance of the Al-10 Vol% Al_2_O_3_-0.25 wt% GO could be attributed to the improvement in the hardness due to the addition of both Al_2_O_3_ and GO particles, with the major contribution coming from the hard Al_2_O_3_ particles. The GO contributed to the reduction in the COF of the hybrid nanocomposite by forming a tribo film over the wear track, as revealed by EDS analysis of the wear track (Figure 6d).

A further increase in GO content (0.5 and 1 wt%) in Al-10 Vol% Al_2_O_3_ matrix resulted in a reduction in hardness, wear resistance and COF. The decrease in the hardness and density of the hybrid samples with the increased amount of GO was attributed to the formation of GO agglomerates, as reported in [44]. The formation of GO agglomerates resulted in a two-phase morphology, leading to a decrease in hardness, which in turn reduced the wear resistance of these hybrid samples (Figure 2). The examination of the SEM images of the wear track, performed after the wear test, indicated the primary wear mechanism to be abrasive wear due to ploughing, as groove marks were visible in the direction of sliding (Figure 6e,f). The scar marks on the counterface balls were also more significant than the balls that slid against the other samples (Figure 7e,f).

## 4. Summary and Comparative Analysis

Table 3 summarizes the results obtained in the current study for all tested samples in terms of relative density, hardness, wear rate and coefficient of friction. Clearly, Al-10 Vol% Al_2_O_3_-0.25 wt% GO nanocomposite showed the highest reduction of 55.65% in specific wear rate compared with Al.

Furthermore, the tribological performance of the Al-10 Vol% Al_2_O_3_-0.25 wt% GO sample showed the lowest wear rate in the current study, as compared with the results reported in the literature. Table 4 compares the tribological performance of the aluminum composites reinforced with Al_2_O_3_ and graphene, as reported in the literature, with the hybrid nanocomposite developed in the current study. It is worth noting that the developed aluminum hybrid nanocomposite reinforced with both Al_2_O_3_ and GO showed excellent tribological performance as compared with the performance of the composites reported in the literature.

## 5. Conclusions

Aluminum (Al) hybrid nanocomposites were fabricated using the spark plasma sintering technique. Alumina (Al_2_O_3_) and graphene oxide (GO) were used to reinforce the Al matrix. The tribological performance of the developed nanocomposites was evaluated. The following conclusions can be drawn from the study:

- Al-10 Vol% Al_2_O_3_-0.25 wt% GO hybrid nanocomposite showed the maximum increase in hardness of 48.4% compared with Al. This significant increase was attributed to the inherent hard nature of Al_2_O_3_ nanoparticles and the uniform dispersion of both the reinforcements within the Al matrix.
- Al-10 Vol% Al_2_O_3_-0.25 wt% GO hybrid nanocomposite showed the lowest specific wear rate. It exhibited the highest reduction of about 55.6% in the specific wear rate as compared with Al. This was attributed to the enhancement in the mechanical properties due to the reinforcements.
- The most predominant wear mechanism was found to be abrasive wear due to plastic deformation, with smooth surface characteristics of the wear track with significantly fewer debris particles. The scar mark on the counterface ball sliding against the Al-10 Vol% Al_2_O_3_-0.25 wt% GO hybrid nanocomposite was also the smallest.

## Figures and Tables

**Table 1:** Spark plasma sintering parameters used for fabricating the hybrid composite samples.

Die Material	Graphite
Die diameter	20 mm
Temperature	550 °C
Heating rate	200 °C/min
Holding time (t)	10 min
Pressure (Pr.)	50 MPa

**Table 2:** Densification of all the sintered samples.

Sample	Relative Density (%)
Al	99.8
Al-10 Vol % Al_2_O_3_	99.5
Al-10 Vol % Al_2_O_3_-0.25 wt% GO	98.9
Al-10 Vol % Al_2_O_3_-0.5 wt% GO	98.6
Al-10 Vol % Al_2_O_3_-1 wt% GO	97.4

**Table 3:** Summary of the results obtained in the current study.

Property	Sample					
	Al	Al + 0.25 wt% GO	Al + 10 Vol% Al_2_O_3_	Al + 10 Vol% Al_2_O_3_+ 0.25 wt% GO	Al + 10 Vol% Al_2_O_3_+ 0.5 wt% GO	Al + 10 Vol% Al_2_O_3_+1 wt% GO
Hardness (HV)	32.8	35.76	55.8	63.56	56.9	56
Relative density (%)	99.8	99.2	99.5	98.9	98.6	97.4
Specific Wear Rate (10^−6^ mm^3^/Nm)	566	439	303	251	837	1920
Reduction in Wear Rate (%)	0	22.63	46.37	55.65	−47.87	−238.8

**Table 4:** Comparative analysis of the results reports in the literature related to Al-based composites.

Composite	Fabrication Method	Relative Density (%)	Hardness	Wear	Reduction in Wear Rate %	Ref
Al-6082	Stir Casting	98.3	74 BHN	62 µg	0	[45]
Al-6082 + 10% Al_2_O_3_		97.5	78 BHN	45 µg	27.41%	
Al-6082 + 15% Al_2_O_3_		97.3	81 BHN	35 µg	43.35%	
Al-6082 + 20% Al_2_O_3_		96.9	87 BHN	30 µg	51.61%	
Al 6061	Stir Casting	−−	95 HV	0.035 gm	0	[46]
Al 6061 + 6% Al_2_O_3_		−−	105 HV	0.028 gm	20%	
Al 6061 + 9% Al_2_O_3_		−−	150 HV	0.023 gm	34.28%	
Al-6061 + 12% Al_2_O_3_		−−	188 HV	0.020 gm	42.85%	
Al-6061	Stir Casting	−−	28 BHN	1.4 × 10^−3^ (mm^3^/m)	0	[47]
Al-6061-5% Al_2_O_3_		−−	33 BHN	0.9 × 10^−3^ (mm^3^/m)	35.71%	
Al-6061-10% Al_2_O_3_		−−	38 BHN	0.64 × 10^−3^ (mm^3^/m)	54.42%	
Al-6061-15% Al_2_O_3_		−−	39 BHN	0.66 × 10^−3^ (mm^3^/m)	52.85%	
Al	Powder Metallurgy	98	111 HV	0.006 (g)	0	[48]
Al + 0.1 wt% GNP		98.8	98 HV	0.005 (g)	16.66%	
Al + 1 wt% GNP		98.8	97 HV	0.007 (g)	−16.66%	
Al 7075	Spark Plasma Sintering	99	96.8 HV	0.0034 (mm^3^/m)	0	[49]
Al-7075/GNPs		99	124.9 HV	0.00275 (mm^3^/m)	19.11%	
A356	Stir and squeeze casting	−−	−−	44 Vol loss (mm^3^)	0	[50]
A356-0.5% Al_2_O_3_		−−	−−	27 Vol loss (mm^3^)	38.63%	
A356-1% Al_2_O_3_		−−	−−	25 Vol loss (mm^3^)	43.18%	
A356-1.5% Al_2_O_3_		−−	−−	32 Vol loss (mm^3^)	27.27%	
Al	Sintering Process	−−	53 HRB	0.039 (g)	0	[51]
Al + 0.01 wt%Greaphene		−−	54 HRB	0.037 (g)	5.12%	
Al + 0.5 wt%Greaphene		−−	62 HRB	0.021 (g)	46.15	
Al + 1 wt%Greaphene		−−	58 HRB	0.055 (g)	−41.02%	
Al + 2 wt%Greaphene		−−	49 HRB	0.105 (g)	−169.23%	
Al + 5 wt%Greaphene		−−	45 HRB	0.170 (g)	−335.89%	
AA 6061	Microwave sintering	−−	65 HV	0.075 (gms)	0	[52]
AA 6061 + 0.3%Graphene		−−	77 HV	0.062 (gms)	17.33%	
AA 6061 + 0.6%Graphene		−−	75 HV	0.062 (gms)	17.33%	
AA 6061 + 0.9%Graphene		−−	73 HV	0.066 (gms)	12%	
AA 6061 + 1.2%Graphene		−−	71 HV	0.070 (gms)	6.66%	
Al-2024	Stir Casting	−−	−−	0.109 (g)	0	[53]
Al-2024 + 0.25 wt% Graphene		−−	−−	0.104 (g)	4.58%	
Al-2024 + 0.50 wt% Graphene		−−	−−	0.0998 (g)	8.44%	
Al-2024 + 0.75 wt% Graphene		−−	−−	0.0942 (g)	13.57%	
Al-2024 + 1 wt% Graphene		−−	−−	0.0892 (g)	18.16%	
Al-0.25wt% GO-10Vol% Al_2_O_3_	**Spark Plasma Sintering**	**98.9**	**63.56 HV**	**251 × 10^−6^ mm^3^/Nm**	**55.65%**	**Present Study**

## Data Availability

Not applicable.
